# Patient-reported outcome measures to deliver patient and family-centered care in pediatrics: the ball is now in our court

**DOI:** 10.3389/frhs.2025.1529731

**Published:** 2025-02-18

**Authors:** Sumedh Bele, Maria J. Santana

**Affiliations:** ^1^ The Nuffield Department of Orthopaedics, Rheumatology and Musculoskeletal Sciences, Medical Sciences Division, University of Oxford, Oxford, United Kingdom; ^2^ Departments of Pediatrics and Community Health Sciences, Cumming School of Medicine, University of Calgary, Calgary, AB, Canada

**Keywords:** patient-reported outcomes, patient-reported outcome measures (PROM), implementation in clinical care, paediatric care, considerations about implementating PROMs

## Abstract

Patient-reported Outcome Measures (PROMs) are self-reported questionnaires that are used to deliver patient and family-centered care, but their use in routine pediatric clinical care remains limited. The American Institute of Medicine (IOM) recommends patient-centered care to achieve high quality health care delivery and PROMs can be used in routine pediatric clinical care to support six critical dimensions of patient-centered care endorsed by the IOM. The growing evidence including our systematic review of pediatric PROMs, shows that incorporating PROMs into routine pediatric clinical practice significantly enhances the quality of care and has a positive impact on Health-related Quality of Life (HRQL) among children and youth. Thus, we are sharing our perspectives on the current evidence, emphasizing the need for using PROMs in routine pediatric clinical care and proposing strategies for pediatric implementation.

## Introduction

The United Nations Convention on the Rights of the Child and the Rights of Persons with Disabilities advocates that children themselves should report their health outcomes whenever possible and should be shown the same respect for personal autonomy accorded to adults when invited to complete questionnaires about themselves (1). Such self-completed questionnaires about outcomes that matter most to children and youth include patient-reported outcome measures (PROMs) (2). As child health services researchers with expertise in investigating the use of PROMs in routine clinical care, we would like to highlight the need for using PROMs in routine clinical care, summarize the evidence on using PROMs in clinical care and recommend strategies to implement PROMs in routine pediatric clinical practice. This article serves as a call to action for all the stakeholders at different levels within pediatric health systems to recognize the significance of using PROMs in routine pediatric clinical care.

PROMs are validated questionnaires that are used to measure individual patient-reported outcomes, and are described as “*any report of the status of a patient's health condition that comes directly from the patient, without interpretation of the patient's response by a clinician or anyone else….*.” (3). Currently, clinicians mostly rely on biological outcomes to evaluate clinical management plans and measure the treatment outcomes. However, despite PROMs' ability to capture a holistic view of well-being and quality of life including physical, functional, psychological and emotional health, as well as physical symptoms like fatigue, pain, and nausea, healthcare providers do not utilize them.

PROMs are scientifically developed and validated questionnaires that can be either generic or disease-specific, designed to evaluate the effects of a disease and/or its treatment from the patient's perspective (4). Children and adolescents living with chronic diseases often experience compromised physical and mental health, along with lower social functioning compared to their healthy peers, which hinders their ability to fully integrate with their peer group (5). As a result, they may feel isolated and excluded (6). Therefore, integrating PROMs into routine pediatric clinical care plays a critical role in improving clinical management by identifying outcomes that matter most to patients and their family caregivers, while also supporting the delivery of patient and family-centered care.

The use of PROMs in pediatric clinical care is limited by the way outcomes are measured and the modes of delivery of care, such as in-hospital vs. community or specialty-based care could facilitate or limit the use of PROMs in clinical care (7). Self-reported outcomes obtained from children and youth are influenced by the importance of friends, peer pressure and school experience. These factors are critical to consider as they shape their aspirations for the future. In pediatric clinical care, outcome measurement is also influenced by the dependency of children on their families and caregivers, therefore proxy reports are widely used (7).

PROMs aim to capture the patient's voice, but younger children or individuals with limited literacy or communication abilities may face challenges in completing these independently. Thus, alternative approaches for completing PROMs include: (1) Proxy reporting; (2) Interview-based methods; (3) using interactive technology-based tools; (4) Using visual analogue scales (VAS) and symbols. While proxies such as caregivers completing the PROMs on behalf of the children add a valuable insight, proxy reporting may not reflect the child's perceptions, as proxies often emphasize observable symptoms over internal experiences (e.g., emotional distress) and tend to overestimate child-reported outcomes. Hence, more efforts are needed to incorporate the assessments of patient-self reports to complement clinical outcomes in routine pediatric care (8).

For younger children, healthcare providers or researchers can administer PROMs through structured or semi-structured interviews with the child. These interviews can be framed conversationally to ensure comprehension. In cases where the children have cognitive challenges and communication is limited or lack of literacy, the use of digital health like tablet applications, computer programs, or gamified interfaces make PROMs more engaging and easier to complete for children. These tools can include pictures, animations, or audio assistance to improve understanding and participation. Furthermore, for children with literacy challenges, audio-assisted PROMs allow them to hear questions and respond by selecting options visually. In younger groups, five years old and younger, tools like smiley faces, emojis, or pictorial scales can be used to help them. For example, the Wong-Baker FACES scale is widely used to measure pain intensity in pediatric care (9). This method simplifies complex questions and makes them developmentally appropriate.

## Role of PROMs in high-quality pediatric clinical care

Although evidence suggests that children as young as eight years old have the cognitive and socio-emotional skills to complete self-reported PROMs, the validity of results produced by PROMs used in pediatrics has been limited by the perceived skepticism about the ability of children and youth to accurately complete such measures (10). Our systematic review also showed that integrating PROMs could have a positive impact on health-related quality of life (HRQL) in the pediatric population (11).

The method chosen to integrate PROMs into clinical care should prioritize accurately capturing the child's perspective and be age-appropriate. Specifically, PROMs should be designed with language and content that is appropriate to the child's developmental stage and cognitive abilities. Considering using multiple approaches—such as self-reports (where possible) supplemented by proxy reports—can provide a more holistic view of the child's experiences. Thus, PROMs are adapted based on the developmental stage, cognitive ability, and communication skills of the child. Table 1 presents some examples:

**Table 1:** Examples of PROMs for different age groups.

Age Group	Examples of PROMs
0–5 years	PROMIS Parent Proxy (12), FLACC Scale (13), Wong-Baker FACES (9) (proxy/visual)
5–7 years	PedsQL Parent Proxy/Child Self-Report (14), Kiddy-KINDL (15), Wong-Baker FACES (9) (simplified)
8–12 years	PedsQL Child Report (14), PROMIS Pediatric (12), FPS-R (16), CHQ (17)
13–18 years	PedsQL Teen Report (14), PROMIS Pediatric, KIDSCREEN-27 (18), YQOL (19)

PROMIS, patient-reported outcomes measurement information systems; FLACC, face, legs, activity, cry, and consolability scale; FPS-R, faces pain scale—revised; CHQ, child health questionnaire; YQOL, youth quality of life.

The Institute of Medicine (IOM), now called the National Academy of Medicine (NAM), is an independent, nonprofit organization in the United States. It was established in 1970 as part of the National Academies of Sciences, Engineering, and Medicine. The IOM provides evidence-based advice to improve health and healthcare through research, policy recommendations, and expert guidance. Its work focuses on a wide range of issues, such as healthcare quality, equity, access, and medical ethics. According to IOM, the implementation of PROMs in pediatric care aligns closely with the six aims for improving healthcare. These six aims are: care that is safe, effective, patient-centered, timely, efficient, and equitable. While the IOM was established in the United States, its six aims for healthcare improvement transcend national boundaries, making them globally applicable. Relation of PROMs to each aim is discussed below:

1. **Safe**: PROMs could help clinicians identify patient symptoms (e.g., pain, fatigue, nausea) and psychosocial issues that may otherwise go unnoticed. Early identification prevents harm by addressing unmet needs, improving symptom management, and ensuring safe treatment plans tailored to the individual.
2. **Effective**: The use of PROMS in clinical care add patient outcomes that inform the effectiveness of treatments (20). PROMs can ensure that therapies are evaluated not just by clinical indicators, but also by improvements in the patient's HRQL and well-being. All in all, the use of PROMs in routine care lead to evidence-based, outcome-driven care (21, 22).
3. **Patient-centered**: PROMs embody the essence of patient-centered care by directly incorporating the patient's voice into clinical decision-making. They facilitate communication between patients and clinicians which may enable healthcare providers to understand what matters most to the child and their family, ensuring care plans align with their needs, preferences, and values (23–25).
4. **Timely:** “PROMs can be collected at various points in time”. Collection of PROMs before the clinical encounter may help early detection of patient's physical, emotional, or social concern. Collecting PROMs after clinical encounter can help monitor patients’ progress and intervene in a timely manner. In fact, PROMs are increasingly being used for remote post-surgical monitoring, allowing surgeons to detect any deteriorations remotely and address them before patients actively seek any assistance (26). Proactive identification of physical, emotional, or social concerns are especially critical in pediatric care where development and disease progression can be rapid.
5. **Efficient:** PROMs can streamline care by focusing attention on the most pressing concerns reported by the patient. By prioritizing interventions that improve patient-relevant outcomes, PROMs could reduce unnecessary testing or treatments that may not address the child's needs, optimizing resource allocation.
6. **Equitable:** IOM's goal on equitable care states that it is important to provide care that does not vary in quality because of personal characteristics such as gender, ethnicity, geographic location, and socioeconomic status. PROMs are standardized tools designed to capture the experiences of all patients. However, standardization overlooks equity concerns arising during development, implementation and interpretation of PROMs (27). One of our studies have found that limited digital literacy, language barriers, discomfort discussing sensitive topics, concerns about impacting care, time constraints, and potential stigma are associated with reporting certain health issues (28). Other well recognized inequities include low socioeconomic status, and disparities based on racial and ethnic identities. While PROMs have the potential to promote equitable care in an ideal, equitable world, the reality is that our societies remain deeply inequitable. Therefore, more health services research is required to make sure PROMs support equitable care to every section of the society (29).

In pediatric clinical care, PROMs can also be used to support six critical dimensions of patient-centered care endorsed by IOM (30), which states that care must be:

(1) Respectful to patient's values, preferences, and expressed needs: Pediatric patients and their family members are most knowledgeable about whether care aligns with their values, preferences, and needs (30). Thus, PROM scores help to measure this dimension of patient-centeredness by facilitating discussions and bringing awareness on patients' views about their values, preferences, and needs.
(2) Coordinated and integrated: Since pediatric patients might be seeking care across various health settings involving several healthcare professionals, it is difficult for the clinicians to determine whether overall patient care was coordinated and integrated (30). Therefore, PROMs could help capture patients' and their families’ perspectives of the delivery of coordinated and integrated care (31, 32).
(3) Provide information, communication, and education: In pediatric clinical care, parents/family caregivers are considered primary agents of care delivery, so questions and information from the medical team are usually directed at them. Therefore, PROMs could support the exchange of appropriate information, communication, and education from pediatric patients' and their families' perspectives (30, 33).
(4) Ensure physical comfort—Only pediatric patients can report on the physical symptoms, such as pain and subjective symptoms, such as fear or anxiety. In some disciplines (e.g., cancer care), PROMs are even recognized as the gold standard for assessing physical comfort (30). Therefore, PROMs could determine if the treatment appropriately attends to patients' physical comfort, physical and emotional symptoms and measure this aspect of patient-centeredness in pediatric clinical care.
(5) Provide emotional support: PROM assessment informs patient-centered care by uncovering children and families' emotional concerns and worries, which often differ from clinicians (30). PROMs can measure whether the emotional support provided during the care is adequate.
(6) Involve family and friends: As mentioned earlier, for pediatric patients, their families are their strengths and support, therefore involving families in pediatric care is highly recommended (34). Although fewer PROMs directly measure this aspect of patient-centered care, similarities and differences in the results between pediatric patients and their family members' PROM results could help understand whether families were adequately involved as the members of the care team.

At the health system level, PROMs can be used to support organization-wide transformation towards patients and family-centered care by incorporating patient-self report outcomes as new data points to healthcare systems that are continuously transforming. To-date, there are several examples that could illustrate the transformations not only at patient level but also at system level. In the United Kingdom, the National Health Service (NHS) routinely collects PROMs for patients undergoing elective procedures such as hip and knee replacements to support patient and family-centered care and policy decisions (35). Similarly, the Danish National PROMs Program routinely collects PROMs nationally for specific conditions like diabetes, cancer, and heart disease (36). In addition, the Amsterdam PROM Implementation Strategy is an example of transformations at the level of integrated care as PROMs are available to providers via their electronic health records (EHR) across clinical settings (37). Likewise, in Canada, specifically in our asthma PROMs' program at the Alberta Children's Hospital, PROMs are available to patients, their family-caregivers and clinicians via an electronic platform named KidsPRO (38). Furthermore, existing evidence highlights that the implementation of PROMs in routine clinical care transforms healthcare systems while informing health policy decisions on healthcare coverage, such as the provision and reimbursement of healthcare services (21, 35–38).

However, the benefits of using PROMs in delivering patient-centered care are contingent on several factors, such as whether the practice environment supports their implementation. The conceptual framework developed by Santana et al. lays the foundation to practice patient-centered care (PCC) (39). This framework classifies key PCC domains according to the Donabedian model for health care development into “structures”, “processes”, and “outcomes”. The *structures* include domains related to the healthcare system or the context in which care is delivered. *Processes* include domains related to patient-health-care-providers interactions. *Outcomes* include domains related to access to care and the use of PROMs (39). For delivering patient and family-centered care, PROMs not only act as direct “outcomes”, but they also have an indirect impact on several other domains within “structures” and “processes”. Traditional objective tests, such as physiological measurements and healthcare utilization metrics, are typically used to assess the impact of a disease on a child, but evidence indicates that the correlation between symptoms and PROMs is stronger than that between symptoms and objective tests (40). PROMs can be used as “pre-assessment” tools to understand patients' needs before their encounter with healthcare system. These patient-reported outcome results can complement other objective test results to get a holistic understanding of patient needs, which helps in shared-decision making and guide treatment plans. PROMs can also be used to monitor treatment over time. This emphasis on patient needs at the core of healthcare delivery can facilitate organization-wide transformation towards patient and family-centered care.

## Strategies to implement PROMs in pediatric clinical care

First, we recommend healthcare systems to recognize the importance of PROMs in routine clinical care, then develop infrastructure, guidelines and protocols to support their implementation. At the organization level, healthcare systems can catalyze such change by enacting evidence-based policies to mandate PROMs in routine clinical care. Then, individual departments and units should engage children, youth and caregivers in selecting PROMs that are most appropriate for their clinical practice and their patient needs. Healthcare providers should develop specific protocols for timing of assessment, mode of administration, and designing clinical care pathways for commonly identified psycho-social issues including prompt referrals to other health professionals like social workers and psychologists. In linguistically diverse societies, PROMs should be available in multiple language versions and developing user guides, and creating “how-to” videos could encourage and support patients and families in completing these measures. Educational and training materials should be co-developed with the patients and their caregivers and with the health care professionals. These materials should be tailored to the audiences and training should be integrated into the regular teaching sessions at the clinics. Quality improvement initiatives should evaluate the impact of PROMs on clinical care and identify mechanisms to optimize using PROMs to improve overall care delivery.

Children with neurodevelopmental disabilities face a greater risk of mental health issues compared to the general population (41). Therefore, special considerations are warranted to successfully use PROMs in their clinical care, such as the careful selection of the most appropriate PROM, having different PROM for the same condition, offering different modes of administering these PROMs (e.g., paper-pencil or electronic versions), and facilitating completion of PROMs in a clinical setting if they are unable to do so at home.

## Conclusion

Integrating PROMs into routine pediatric care alone is not enough on its own to ensure patient and family-centered care. However, PROMs can serve as a catalyst for organization-wide changes and play a vital role in transforming clinical care practices in pediatrics. Now is the time to embrace this opportunity to integrate PROMs in pediatric clinical care—the responsibility lies with us.

## Data Availability

The original contributions presented in the study are included in the article/Supplementary Material, further inquiries can be directed to the corresponding author.
